# Healthcare Professionals’ Attitudes and Practices According to Their Recommendations on Exercise during the First Trimester of Pregnancy: A Greek Cross-Sectional Study

**DOI:** 10.3390/sports12070173

**Published:** 2024-06-24

**Authors:** Vasileios Daglas, Nikolaos Kostopoulos, Irina Mrvoljak-Theodoropoulou, Michalis Mitrotasios, Maria Dagla, Aikaterini Lykeridou, Evangelia Antoniou

**Affiliations:** 1 Department of Midwifery, School of Health and Care Sciences, University of West Attica, 12243 Athens, Greece; mariadagla@uniwa.gr (M.D.); klyker@uniwa.gr (A.L.); lilanton@uniwa.gr (E.A.); 2 School of Physical Education and Sport Science, National and Kapodistrian University of Athens, 17237 Athens, Greece; nikkosto@phed.uoa.gr (N.K.); micmit@phed.uoa.gr (M.M.); 3 Department of Psychology, National and Kapodistrian University of Athens, 15784 Athens, Greece; imrvoljak@hotmail.com

**Keywords:** exercise, pregnancy, healthcare professionals, attitudes, practices, first trimester

## Abstract

Background: The aim of this study is to investigate healthcare professionals’ attitudes and practices when it comes to their recommendations on exercise during the first trimester of pregnancy and to highlight the factors that influence or predict these attitudes. Methods: This cross-sectional study was conducted between January 2022 and March 2023, on a sample of 237 Greek healthcare professionals (midwives and obstetricians) employed in healthcare settings in Attica/Greece. In the statistical analysis, eight independent models of multivariate analyses of variance were conducted. Results: Only half of the participants (54.89%) report that they recommend exercise to pregnant women in the first trimester of pregnancy. The majority do not routinely recommend a specific frequency and duration of exercise. Participants who believe that exercise during pregnancy is of little benefit to pregnant women were less likely to recommend the following, in the first trimester of pregnancy: (a) exercise in general (*p* = 0.002), (b) resistance/muscle strengthening exercises (*p* = 0.039), (c) relaxation exercises (*p* = 0.002), and (d) a specific exercise duration (*p* = 0.011). Those who report being very familiar with the international guidelines are (a) more likely to recommend exercise in general (*p* = 0.013), as well as aerobic exercises (*p* = 0.023); (b) less likely to not recommend a specific frequency (*p* = 0.027); and (c) more likely to recommend a duration of 30–45 min of exercise in the first trimester (*p* = 0.017). Conclusions: I this study, a significant proportion of health professionals’ attitudes appear to diverge from the recommendations set forth by international scientific bodies regarding exercise during pregnancy. Furthermore, health professionals’ beliefs regarding the benefits of exercise during pregnancy, along with their familiarity with international guidelines, appear to influence their usual practices in recommending exercise during the first trimester of pregnancy to pregnant women.

## 1. Introduction

Physical activity is a vital component of a healthy lifestyle, contributing to weight control and overall health. During pregnancy, it has been associated with very positive and beneficial effects on maternal health (e.g., reduction in pregnancy weight, risk of gestational diabetes mellitus, risk of pre-eclampsia, need for cesarean section or instrumental labor, anxiety, or perinatal depressive symptoms) [1,2,3,4,5,6]. Despite international recommendations encouraging physical activity during pregnancy (when there is no medical contraindication), many pregnant women either do not follow a physical activity program or severely limit their physical activity [7,8], while there is often a lack of documented data for this subject between countries.

A key parameter directly related to this issue is the recommendations offered to pregnant women by healthcare professionals who are qualified to provide care during pregnancy, i.e., midwives and obstetricians. The role of these healthcare professionals appears to be important [9,10]. Their attitudes and behavior, with regard to providing specific recommendations, advice, and information on the adoption of healthy behavioral patterns, seem to clearly influence the behavior of pregnant women on the issue of exercise [11]. In fact, professionals themselves believe that providing lifestyle advice antenatally and during maternity care would contribute significantly to addressing public health issues (such as the issue of obesity) [12]. Unfortunately, however, there is a lack of evidence in the recent international literature on the recommendations provided by healthcare professionals to pregnant women and the extent to which these are in line with international guidelines. Nevertheless, evidence from previous relevant studies shows that obstetricians do not routinely advise pregnant women who were not exercising or were inactive to start an exercise program, are conservative when it comes to exercise intensity, and tend to advise a reduction in exercise volume in the third trimester, even in healthy pregnancies [13]. It also appears that women receive little or no advice on prenatal physical activity and exercise [10], or that the advice they do receive is unclear and inconsistent [14].

When it comes to the crucial role of midwives in the dissemination of correct and reliable information on exercise during pregnancy [15], most appear to recognize and accept its usefulness during pregnancy and its beneficial effects on both maternal and fetal health [16,17]. However, when looking into the advice they provide, it is found that they do not accurately inform pregnant women about the recommended levels of intensity, frequency, and duration of exercise [18]. Regarding midwives’ views on the safety of exercise and whether they recommend the participation of pregnant women who did not exercise prior to pregnancy, in some cases, they appear reluctant and do not encourage them to take up exercise during pregnancy, even though they largely agree on its safety [16,18]. 

These healthcare professionals consider a lack of training, knowledge, confidence, time, resources, and perceptions of vulnerability as important barriers to promoting physical activity during pregnancy [15]. Among other perceived barriers to the effectiveness of their counseling, midwives report the cultural beliefs of some pregnant women that limit their participation in antenatal exercise programs [19], while they also point to a general absence of community support for pregnant women with the lack of, e.g., available antenatal exercise programs [17], as well as to the beneficial role and motivation that partner support can provide [15]. In a recent study focused on barriers to exercise during the first trimester of pregnancy, findings indicated that factors such as higher education, white race, marital status, and having a normal BMI were associated with meeting international exercise guidelines for pregnant women [20].

Considering that a pregnant woman’s lifestyle can influence the occurrence of complications in pregnancy, her own health, and that of her child [6], and knowing that regular physical activity during pregnancy is associated with many benefits, while at the same time, as has been recently documented, is not associated with an increased risk of maternal or perinatal adverse outcomes and is considered safe for both mother and fetus [6], counseling by any healthcare professional who monitors and cares for pregnant women should include information and guidance on exercise and physical activity. Due to variations among countries in healthcare services, available resources, practices, and cultural factors that can influence health professionals’ attitudes and behavior regarding exercise during pregnancy, investigating this sensitive topic is crucial. Moreover, considering the limited number of recent studies on this subject, exploring healthcare professionals’ attitudes and behavior will contribute new evidence-based knowledge. This investigation is particularly pertinent when examining recommendations for pregnant women in the first trimester, a critical period for several reasons. First, research indicates that many women tend to reduce their exercise levels early in pregnancy [21], often due to concerns about miscarriage, despite recent evidence suggesting no increased risk of miscarriage with low- to moderate-intensity exercise [22]. Second, there is a notable lack of data on the exercise guidelines provided to pregnant women during this early stage of pregnancy. Therefore, studies that highlight health professionals’ attitudes and behavior towards exercise will underscore the importance of this topic and serve as a trigger for the design and implementation of innovative interventions aimed at changing and modifying misguided behavior and, generally, for the promotion of proper healthcare practices and standards in each country.

The aim of this study is to investigate healthcare professionals’ attitudes (midwives and obstetricians) and the practices they follow when it comes to their recommendations on exercise during the first trimester of pregnancy and to highlight the factors that influence or predict these attitudes. 

## 2. Materials and Methods

### 2.1. Study Population

This cross-sectional study was conducted between January 2022 and March 2023 on a sample of 237 Greek healthcare professionals (midwives and obstetricians), who were employed at healthcare facilities (public and private) in the prefecture of Attica, during the period of the study. Healthcare professionals were recruited in two public general hospitals, where most pregnant women, women who are giving birth or have recently given birth, and newborns are monitored and nursed. In addition, midwives and obstetricians working in public primary care facilities of the 1st Regional Health Authority of Attica and providing pregnancy and postnatal counseling and care, as well as healthcare professionals working in the private sector (in one of the largest general/obstetric clinics in the prefecture of Attica) or as freelancers, were recruited in the survey. This research utilized convenience sampling, with the sample drawn from healthcare settings that were readily accessible. This population was chosen because midwives and obstetricians, due to their training and professional experience, are the main providers of healthcare services throughout pregnancy. In addition, these healthcare professionals are required to provide counseling on lifestyle issues for pregnant women, advocate practices that promote the health of pregnant women and the fetus, and detect pathological conditions.

### 2.2. Data Collection 

The eligibility criteria for this study were defined as follows: (a) possession of a midwifery and/or medical school degree (higher education), (b) specialization as an obstetrician or currently undergoing training for the obstetric specialty, (c) a valid license to practice midwifery or medicine, and (d) at least one year of experience in monitoring pregnant women. Initially, 447 health professionals who met the eligibility criteria were approached and informed about the survey (281 midwives and 166 obstetricians). Among them, 237 (153 midwives and 84 obstetricians; response rate 53%) agreed to participate voluntarily and provided written informed consent. The recruited participants were asked to complete an anonymous self-report questionnaire created for the purpose of this study. 

This questionnaire comprised the following sections: (a) socio-demographic and occupational information, (b) healthcare professionals’ views and beliefs on the topic of exercise during pregnancy, (c) practices they usually follow in each trimester of pregnancy, and (d) knowledge of international guidelines on the topic. This article presents the results obtained from the analyses of the data collected from section c and, more specifically, information relating to the practices followed and recommendations provided by midwives and obstetricians during the 1st trimester of pregnancy. In addition, the relationship between these data and their socio-demographic, occupational characteristics, and healthcare professionals’ beliefs and their knowledge about international guidelines on the topic were explored. 

The first nine questions of the questionnaire captured the socio-demographic characteristics of health professionals and details about their professional activity (e.g., gender, age, education level, specialty, experience, professional setting, etc.). In Section C, participants were asked to indicate for each trimester of pregnancy: (a) whether they recommend exercise in general (yes/no), (b) whether they recommend aerobic exercises (if yes, which ones), (c) whether they recommend resistance/muscle strengthening exercises (if yes, which ones), (d) whether they recommend relaxation exercises (if yes, which ones), (e) whether they recommend a specific exercise frequency (if yes, which one), and (f) whether they recommend a specific exercise duration (if yes, which one). Additionally, to explore health professionals’ exercise recommendations in relation to their beliefs about exercise and their knowledge of international guidelines, participants were asked: (a) to what extent they believe exercise during pregnancy is generally beneficial for women (Section B), (b) their perceived level of familiarity with international guidelines/recommendations for exercise during pregnancy (Section D), and (c) to what extent they believe there is a necessity to inform midwives and obstetricians about guidelines on exercise during pregnancy (Section D). Responses to these questions used a 5-point Likert scale ranging from “not at all” to “very much”.

Ethical approval was obtained from the scientific and ethics committees of the three hospitals where the survey was conducted and from the 1st Regional Health Authority (RHA) of Attica [Ref. Number (1st public hospital): 41/20-01-2022, Ref. Number (2nd public hospital): 1480/28-01-2022, Ref. Number (1st Regional Health Authority): 21855/20-05-2022 and Ref. Number (private hospital): 9-12-2022]. Throughout the study, strict adherence to the research code of ethics was maintained. All participants provided written consent to participate in the study, after having been informed orally and in writing about the objectives and the way the study would be conducted. The participating healthcare setting and healthcare professionals were coded, so personal or professional information or any of their data remained anonymous and strictly confidential throughout the study. 

### 2.3. Statistical Analysis

Statistical significance was set at 0.05, and data analyses were performed using the Statistical Package for Social Sciences (SPSS) version 22.0 (SPSS Inc., Chicago, IL, USA). Quantitative variables were described as absolute frequencies (*n*) and relative frequencies (*%*). Healthcare professionals, recommendations to pregnant women on exercise, and, more specifically, the type, frequency, and duration of exercise they recommend were defined as dependent variables. Independent variables included (a) socio-demographic characteristics: gender, age, educational level; (b) information on professional activity: professional specialty, professional experience (total), professional setting (public sector, private sector, primary care facility, freelancer), professional position of physicians (e.g., director, etc.), and midwives’ experience in antenatal counseling programs; (c) healthcare professionals’ belief on the benefits of exercise during pregnancy; (d) their perceived level of familiarity with international guidelines/recommendations for exercise during pregnancy; and (e) the degree of necessity (according to them) to be informed about guidelines for exercise during pregnancy. Analyses were performed using the Chi-square test and Fisher’s Exact Test. Additionally, multiple analyses of variance (ANOVA) were conducted to examine the relationship between the practices followed by healthcare professionals in their recommendations to pregnant women about exercise and several independent variables. Furthermore, binary logistic regression was used to investigate the relationship between participants’ recommendations on the frequency and duration of exercise in the 1st trimester and various independent variables.

## 3. Results

Table 1 presents the demographic and occupational characteristics of the 237 participants of the study. As can be observed, the participants: (a) are mainly female (75.10%, *N* = 178), (b) have an average age of 40.65 years (*SD* = 11.11), (c) are mainly midwives (64.6%, *N* = 153), (d) hold a postgraduate degree (38. 4%, *N* = 91), (e) have an average of 14.53 years of professional experience (*SD* = 9.51), and (f) about half of them work in a private hospital or are freelancers (52.8%, *N* = 125), while the rest work in a public hospital or a public primary care facility (47.3%, *N* = 112). 

Regarding their recommendations to pregnant women on exercise (Table 2), half of the participants claim that they recommend exercising in the first trimester (54.89%, *N* = 129). Also, half of them claim to recommend aerobic exercises (53.19%, *N* = 125) and relaxation exercises (52.77%, *N* = 124) in the first trimester of pregnancy, while only 25.96% (*N* = 61) appear to recommend resistance/muscle strengthening exercises. Most participants do not recommend either a specific frequency (59.9%, *N* = 140) or a specific duration of exercise (59.9%, *N* = 140) in the first trimester. 

**Figure 1:**
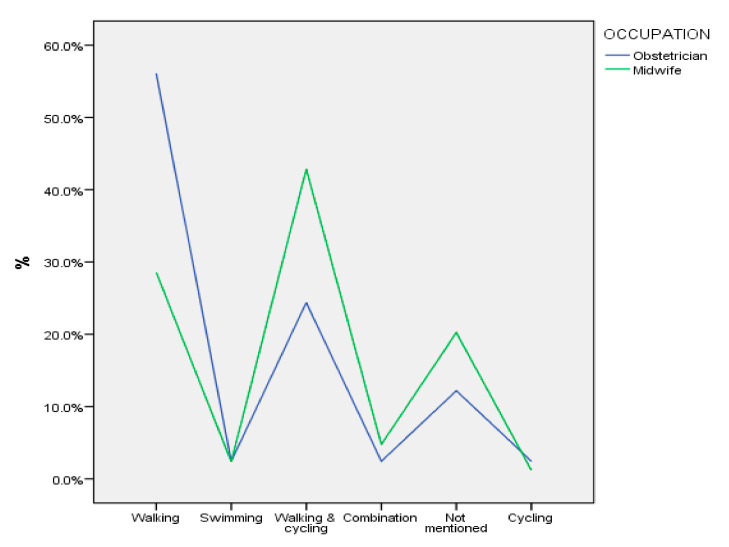
Aerobic exercises recommended by participants in the first trimester of pregnancy.

**Figure 2:**
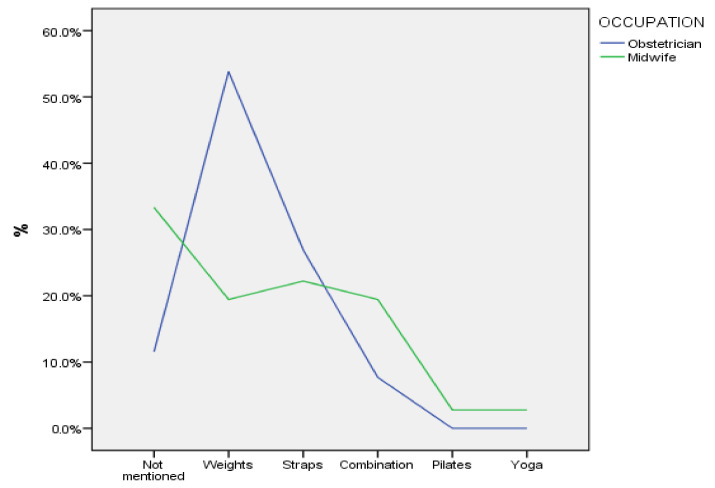
Resistance/strengthening exercises recommended by participants in the first trimester of pregnancy.

**Figure 3:**
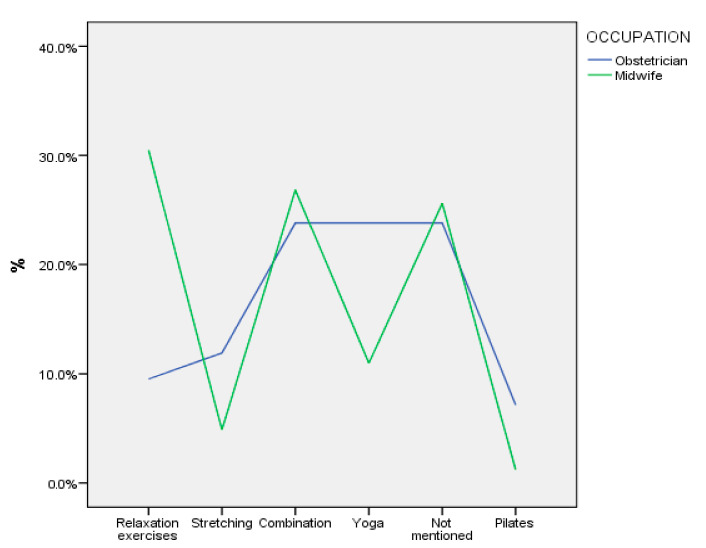
Relaxation exercises recommended by participants in the first trimester of pregnancy.

Figure 1, Figure 2 and Figure 3 illustrate the practices followed by the participants in terms of the type of exercises they recommend in the first trimester of pregnancy, according to their professional occupation. Most obstetricians (>50%) report that they recommend swimming (as an aerobic exercise) and weights (for strength training), while several of them seem to recommend a combination of relaxation exercises or yoga or do not specifically mention any type of relaxation exercise. Midwives recommend a combination of walking and swimming as an aerobic exercise, they do not mention any specific recommendation for strength training (>30%), while several of them appear to recommend relaxation exercises or a combination of exercises.

Table 3 presents the results of the analyses exploring the relationship between participants’ recommendations on exercise in the first trimester of pregnancy and their occupational characteristics. Using the *χ*^2^ statistical test, two independent analyses revealed statistically significant relationships indicating that midwives with experience in antenatal counseling programs were more likely to recommend exercise in general in the first trimester of pregnancy (*p* = 0.034), as well as aerobic exercise (*p* = 0.014), compared to midwives without such experience. Also, the relationship between midwives’ recommendations for resistance/muscle strengthening exercises in the first trimester of pregnancy and their professional position was examined using the Fisher Exact test for three independent samples, revealing a statistically significant relationship between these variables (*p* = 0.018). As shown, freelancers were more likely to recommend resistance/muscle strengthening exercises in the first trimester of pregnancy than other health professionals (Table 3).

Table 4 presents the results of four independent models of multivariate analyses of variance designed to investigate the relationship between healthcare professionals’ recommendations on exercise in the first trimester of pregnancy and several independent variables. In all the following predictive models, only statistically significant predictors are presented. Stepwise regression was used to present only statistically significant relationships. 

Thus, as shown in Model A, men (*p* = 0.024), as well as healthcare professionals who believe that exercise is moderately beneficial for pregnant women (“moderately” was the lowest degree of perceived benefit noted by participants) (*p* = 0.002), tend to recommend exercise in the first trimester of pregnancy to a lesser extent. In contrast, healthcare professionals who reported being “very” familiar with international guidelines/recommendations for exercise during pregnancy were more likely to recommend exercise in the first trimester (*p* = 0.013). This pattern explained 13.2% of the overall variance of exercise recommendation in the first trimester (Table 4).

In the next binary logistic regression analysis model (Model B, Table 4), examining participants’ recommendations of aerobic exercise in the first trimester of pregnancy, the following predictors emerged: (a) professional setting (private sector) (*p* = 0.032), (b) healthcare professionals’ level of familiarity with international guidelines/recommendations for exercise during pregnancy (“very”) (*p* = 0.023), and (c) the level of necessity to be informed about guidelines on exercise during pregnancy (“some”/“moderate”, the lowest levels of necessity noted by the participants) (*p* = 0.008). This pattern of analysis explained 11.4% of the dependent variable. As shown, healthcare professionals (midwives and obstetricians) working in the private sector, and those who believe that there is some/moderate necessity to be informed about guidelines for exercise during pregnancy, recommend aerobic exercise in the first trimester of pregnancy to a lesser extent. In contrast, healthcare professionals who reported being “very” familiar with international guidelines/recommendations for exercise during pregnancy are more likely to recommend aerobic exercises in the first trimester of pregnancy (Table 4).

In Model C (Table 4), the participants’ recommendation of resistance/muscle strengthening exercises in the first trimester of pregnancy was defined as the dependent variable and two variables emerged as predictors: (a) the participants’ professional setting (freelancers) (*p* = 0.039) and (b) whether they believe that exercise during pregnancy is beneficial (“moderate”, the lowest degree of perceived benefit noted by participants) (*p* = 0.039). This model explains 9.1% of the total variance of the dependent variable. As shown, freelancers were more likely to recommend resistance/muscle-strengthening exercises to pregnant women in the first trimester of pregnancy. Also, healthcare professionals who believe that exercise is moderately beneficial to pregnant women were less likely to recommend these exercises in the first trimester of pregnancy.

In the binary logistic regression analysis model D (Table 4), participants’ recommendation of relaxation exercises in the first trimester of pregnancy was used as the dependent variable and the extent to which they believe exercise during pregnancy is beneficial (“moderate” was the lowest degree of perceived benefit noted by participants) was derived as the predictor (*p* = 0.002). This model explained 7.4% of the dependent variable. As shown, healthcare professionals who believe that exercise during pregnancy benefits women to a moderate degree, recommend relaxation exercises in the first trimester of pregnancy to a lesser extent.

Table 5 presents the results obtained from four independent models of multivariate analyses of variance designed to investigate the relationship between healthcare professionals’ recommendations on the frequency and duration of exercise in the first trimester of pregnancy and several independent variables. In Model A (Table 5), in which the participants’ practice of not recommending a specific frequency of exercise to pregnant women in the first trimester of pregnancy was defined as the dependent variable, the predictors that emerged included (a) the professional healthcare setting (freelancer) (*p* = 0.022), (b) whether participants believe that exercise during pregnancy is beneficial (“moderate”, the lowest degree of benefit noted by participants) (*p* = 0.007), and (c) their level of familiarity with international guidelines/recommendations for exercise during pregnancy (“very”) (*p* = 0.027). The model explained 12.7% of the total variance of the dependent variable. As shown, freelancers and those healthcare professionals who report being very up to date with international guidelines/recommendations for exercise during pregnancy were less likely to not recommend a specific frequency of exercise in the first trimester of pregnancy. In contrast, those who believe that exercise is moderately beneficial for pregnant women appear to be more inclined not to recommend a specific exercise frequency.

In the next binary logistic regression analysis model (Model B, Table 5), healthcare professionals’ recommendation of exercise at a frequency of 1 to 3 days/week in the first trimester of pregnancy was used as the dependent variable, while the extent to which they believed that exercise during pregnancy is beneficial (“moderate”—the lowest degree of benefit noted by the participants) (*p* = 0.046) emerged as the predictor. This model explained 3.5% of the total variance of the dependent variable. As shown, participants who believe that exercise during pregnancy is moderately beneficial were less likely to recommend an exercise frequency of 1–3 days/week in the first trimester of pregnancy.

In Model C (Table 5), healthcare professionals’ practice of not recommending a specific duration of exercise to pregnant women in the first trimester of pregnancy was set as the dependent variable. The extent to which professionals believe that exercise during pregnancy is beneficial (“moderate”—the lowest degree of benefit noted by the participants) (*p* = 0.011) emerged as the predictor. This model explains 6.1% of the total variance. Therefore, participants who believe that exercise during pregnancy moderately benefits pregnant women do not tend to recommend a specific duration of exercise in the first trimester of pregnancy.

Regarding the next binary logistic regression model (Model D Table 5), healthcare professionals’ recommendation of 30–45 min of exercise in the first trimester of pregnancy was used as the dependent variable. The predictors identified included (a) professional healthcare setting (public sector) (*p* = 0.009) and (b) participants’ level of familiarity with international guidelines/recommendations for exercise during pregnancy (“very”) (*p* = 0.017). This model explained 21.0% of the total variance. As shown, healthcare professionals working in the public sector and those who report being very familiar with the international guidelines/recommendations for exercise during pregnancy, were more likely to recommend 30–45 min of exercise in the first trimester of pregnancy.

## 4. Discussion

This study highlights healthcare professionals’ (midwives and obstetricians) attitudes and their practices regarding their recommendations on exercise in the first trimester of pregnancy, as well as the factors that influence or predict these attitudes. Half of the midwives and obstetricians who participated in the survey (54.89%) reported that they recommend exercise to pregnant women in the first trimester, mainly aerobic and relaxation exercises rather than resistance/muscle strengthening exercises. Our results are consistent with those of other studies [7,13,17] demonstrating that exercise counseling for pregnant women is provided in approximately half of the cases, and aerobic exercise usually seems to be preferred over resistance exercise. Furthermore, the proportion of participants in this study recommending exercise during pregnancy appears to be sometimes higher than in other studies [23,24], in which only a quarter of respondents seem to discuss exercise with most pregnant women, and sometimes lower, e.g., when compared with the results of the Santo et al. study [25]. 

This study also demonstrated, for the first time in the international literature, that healthcare professionals’ beliefs about the benefits of exercise during pregnancy, their level of familiarity with, and the necessity to be informed about, international guidelines for exercise during pregnancy are the key predictors of which recommendations they provide pregnant women in the first trimester. Although it is known that healthcare professionals believe that exercise during pregnancy is beneficial [23,26,27,28], it remains unclear to what extent these beliefs influence their behavior in terms of the practices they follow. Also, although we were aware that healthcare professionals have little or no scientific knowledge of the guidelines for exercise during pregnancy [29,30], the extent to which this influenced their practice was not known. 

In this study, it appears that healthcare professionals who believe that exercise during pregnancy is of little benefit to pregnant women were less likely to recommend the following, in the first trimester of pregnancy: (a) exercise in general, (b) resistance/muscle strengthening exercises, and (c) relaxation exercises. Furthermore, those who reported being very familiar with the international guidelines/recommendations for exercise during pregnancy were more likely to recommend exercise in general, and aerobic exercises in the first trimester of pregnancy, while those who believed there was little or moderate need to be informed were less likely to recommend aerobic exercises during that period.

Two recent studies, which assessed the knowledge, attitudes, beliefs, and recommendations of obstetricians (in the USA) [23] and medical practitioners (in South Africa) [27], have concluded that (a) only a quarter of obstetricians appear to regularly discuss exercise issues with pregnant women, while most do not; (b) a quarter do not recommend strengthening exercises in the first trimester; (c) several recommend reducing aerobic and strengthening exercises in the third trimester [23]; and (d) the vast majority of medical practitioners appear to be unaware of guidelines for exercise during pregnancy [27]. Similarly, in another study, several healthcare professionals reported being unsure whether the information they provide to pregnant women about antenatal exercise is in line with current guidelines [28]. 

Midwives seem to understand their professional responsibility to advise and guide pregnant women regarding exercise and physical activity [15,31]. In a study that investigated the barriers to and facilitators of physical activity among obese pregnant women, and the application of international guidelines by midwives, the main conclusion was that midwives seem to have the required knowledge about the necessity and value of physical activity counseling for obese pregnant women and consider this area to be part of their professional role [31]. However, they report perceiving themselves as lacking both the necessary skills and resources, and that they do not plan or prioritize discussions with obese pregnant women on this topic [31]. 

The findings of this study contradict the view reported in a previous study [23] suggesting that the lack of counseling on the part of healthcare professionals, specifically obstetricians, is not likely to be due to (a) misconceptions on their part about the benefits of exercise during pregnancy, and (b) misinterpretation of guidelines by scientific bodies. The authors support this view based on the fact that the vast majority of the sample of obstetricians they surveyed claimed that exercise is associated with benefits such as the prevention of excessive weight gain (91%) and a decreased risk of postpartum depression (77%), and because almost all of them (98%) claimed to believe that the benefits of exercise in uncomplicated pregnancies outweigh any potential risks. 

Similarly, to that study [23], in our study, the vast majority of participants (88.6%) report believing that exercise during pregnancy is generally beneficial (these data are not presented in the results). Nevertheless, logistic regression models applied in this study indicated that where healthcare professionals believe that exercise during pregnancy is beneficial to a small degree, they tend to recommend it less in the first trimester of pregnancy. Therefore, although healthcare professionals’ beliefs about the benefits of exercise during pregnancy are largely positive, their lack of knowledge about exercise in general and about the guidelines in particular may contribute to their reluctance and lack of active involvement in providing recommendations and antenatal advice to pregnant women. It is possible that healthcare professionals feel uncertain about what is appropriate or safe, are not aware of accurate details about exercise during pregnancy (type, frequency, intensity, duration), and, therefore, do not often provide counseling and do not recommend it. Moreover, they themselves admit [15,17,30] that they are not equipped to provide effective counseling and guidance on physical activity for pregnant women. This could explain why the majority of healthcare professionals do not routinely recommend a specific frequency or duration of exercise, a fact that has been documented by several studies [27,32] and confirmed by our study. In addition, women report that they receive recommendations from healthcare professionals that are inconsistent with guidelines, inadequate, or incorrect [15,18,23,27,30]. 

At the same time, the present study shows that participants who believe that exercise during pregnancy is of little benefit: (a) are less likely to recommend a frequency of exercise of 1–3 days/week, and (b) do not tend to recommend a specific duration of exercise in the first trimester of pregnancy. Furthermore, their level of familiarity with international guidelines seems to predict their behavior regarding their recommendations. Thus, those who report being very familiar with the guidelines: (a) are less likely to not recommend a specific exercise frequency—a practice that freelancers also seem to follow less; and (b) are more likely to recommend a duration of exercise of 30–45 min in the first trimester—a practice that also appears to be followed by those who work in the public sector. 

Women are more likely to exercise during pregnancy following their doctor’s recommendations [23,33,34] because of the well-established influence of healthcare professionals on them. Considering the fact that (a) healthcare professionals themselves claim to often rely on common sense or their own experience when providing counseling and guidance to pregnant women on antenatal exercise [15,30], (b) they pursue education and seek new tools to improve their techniques in providing counseling to pregnant women on issues such as weight gain [35], and (c) there is evidence [36] that interventions aimed at educating healthcare professionals can improve their knowledge and practices relating to health issues in the perinatal period; the findings of our study reaffirm the importance of informing and educating healthcare professionals about exercise and its benefits during pregnancy, but also indicate the great necessity for training and skills development of healthcare professionals, as has been highlighted and discussed in previous studies [15,17,18,19,22,23,24,25,26,27,28,29,30,37,38]. They also encourage the implementation of public health interventions and policies aimed at healthcare professionals who provide counseling to women of childbearing age, in order to promote the adoption of an active lifestyle during pregnancy, so as to avoid sedentary lifestyles and reduce the risks associated with obesity [7].

At the same time, the need for adequate training for healthcare professionals providing counseling to pregnant women on exercise becomes imperative, knowing that (a) pregnant women are inactive or less active than non-pregnant women, with the prevalence of physical activity being particularly low during pregnancy [7], especially in the first and third trimester [7]; (b) women who self-identify as inactive tend to become more sedentary and less physically active as pregnancy progresses [8], and many women who were physically active before pregnancy discontinue or reduce their activity levels once pregnancy begins [7,39]; and (c) increased activity in early pregnancy is associated with a higher level of physical activity later in pregnancy [40]. Therefore, proper training of healthcare professionals and the adoption of standardized protocols for exercise during pregnancy, such as those from the World Health Organization and the National Institute for Health and Clinical Excellence, which are considered to have the highest methodological quality [41], appear to be essential.

In a recent study designed to evaluate the effect of an online educational intervention (exercise instructions, brief counseling methods, and exercise programs) on physicians’ attitudes, beliefs, knowledge, and practices regarding antenatal exercise, improvements in several factors were observed [29]. Upon completion of the intervention, obstetricians’ knowledge of international guidelines for exercise during pregnancy, their ability to recommend appropriate and safe exercises, and their familiarity with exercise programs were significantly improved. Training will contribute to the minimization of misconceptions associating exercise with complications in low-risk pregnancies, will reinforce the belief that exercise is a valuable way to enhance the health of pregnant women and the fetus, and will promote the implementation of international guidelines. Training programs should aim to familiarize healthcare professionals with current guidelines, enrich and update their knowledge, and help them develop the necessary skills to correct misconceptions and attitudes that may pose risks to maternal and fetal health.

Considering the lack of recent international research on factors that predict healthcare professionals’ practices regarding the provision of exercise recommendations to pregnant women in the first trimester of pregnancy—which are likely to determine the behavior of pregnant women in the following trimesters as well—such studies bring to light unexplored relationships and interactions that are of particular value. The results of the present study should be considered when designing and implementing interventions and programs aimed at promoting exercise during pregnancy in general, and more particularly, in cases of pregnant women who were either inactive before pregnancy or are overweight in pregnancy, a pathological condition that complicates pregnancy and is responsible for short- and long-term consequences for both the mother and the offspring [42]. Such targeted education programs for healthcare professionals are of particular value in countries such as Greece, where exercise rates are low and obesity rates are high in the general population [43,44]. 

A limitation of this study is its sample, which does not represent all midwives and obstetricians in the country. In order to collect the recommendations of both specialties, we approached midwives and obstetricians working in both the public and private sectors and in every level of health care (primary and secondary/tertiary). We chose to approach healthcare professionals employed in two large public hospitals with the highest attendance of pregnant women in the prefecture of Attica and in a large private hospital that also receives a high number of pregnant women in the same region, as well as in all the primary care facilities under the 1st Regional Health Authority of Attica. Furthermore, considering that most pregnant women are monitored in hospitals in Attica, there is a lot of experience among the specific healthcare professionals we approached, and having recruited a large number of participants, we can argue that our sample is suitable and represents to a satisfactory extent the views of midwives and obstetricians in the country. 

Another limitation of this study is that our data were obtained based on healthcare professionals’ self-reported practices and, thus, may not fully represent their actual practices. However, it should be emphasized that the participants were asked to answer the questions based on their own routine behavior rather than what is generally considered acceptable. They were, also, assured that their participation was confidential and anonymous and that their personal and workplace details would not be made public. It is also worth mentioning that investigating the self-reported practices of practitioners is a common practice in international surveys, and many conclusions of international surveys are drawn using such methodologies. 

## 5. Conclusions

The findings of the present study lead to a better understanding of healthcare professionals’ (midwives and obstetricians) behavior in providing care and counseling to pregnant women when it comes to their recommendations on exercise in the first trimester of pregnancy and the factors that are associated with or may predict this behavior. As can be concluded, only half of the healthcare professionals state that they recommend exercise to pregnant women in the first trimester of pregnancy, in particular, aerobic exercise and relaxation exercises. The majority do not routinely recommend either a specific frequency or a specific duration of exercise in the first trimester. The attitudes and beliefs of midwives and obstetricians regarding the benefits of exercise during pregnancy in general, their familiarity with the guidelines on this subject, and the perceived degree of necessity to be informed appear to be the key factors determining the recommendations they will provide to pregnant women in the first trimester regarding exercise in general and, in particular, the type, frequency, and duration of exercise. Therefore, the results of this study are useful for the systematic design of sound policies and interventions aimed at reversing misconceptions and harmful attitudes and behavior related to women’s health that negatively affect perinatal outcomes, and, at the same time, contribute to the adoption of proper practices on the part of healthcare professionals that will promote the health of the woman and her child. 

## Figures and Tables

**Table 1:** Participants’ socio-demographic and occupational characteristics.

	*N/M*	*%/SD*
Gender		
Male	59	24.9
Female	178	75.1
Total	237	100.0
Age	40.65	11.11
Occupation		
Obstetrician	84	35.4
Midwife	153	64.6
Total	237	100.0
Educational level		
Undergraduate	127	53.6
Postgraduate	91	38.4
Doctorate	18	8.0
Total	236	100.0
Work experience (years)	14.53	9.51
1–5 years	42	19.0
6–10 years	57	25.8
11–15 years	32	14.5
16–20 years	32	14.5
>20 years	58	26.2
Total	221	100.0
Professional healthcare setting		
Public hospital	72	30.4
Public primary care facility	40	16.9
Private hospital	31	13.1
Self-employed	94	39.7
Total	237	100.0

**Table 2:** Practices followed by healthcare professionals regarding their recommendations on exercise in 1st trimester of pregnancy.

	*N*	*%*
Recommend exercise		
No	106	45.11
Yes	129	54.89
Total	235	100.0
Recommend aerobic exercises		
No	110	46.81
Yes	125	53.19
Total	235	100.0
Recommend resistance/muscle strengthening exercises		
No	174	74.04
Yes	61	25.96
Total	235	100.0
Recommend relaxation exercises		
No	111	47.23
Yes	124	52.77
Total	235	100.0
The frequency of exercise they recommend		
No specific recommendation	140	59.9
1 day	5	2.1
2–3 days	67	28.3
4–5 days	9	3.8
5–6 days	3	1.3
Everyday	11	4.6
Total	235	100.0
The duration of exercise they recommend		
No specific recommendation	142	59.9
15–30 min	41	17.7
30–45 min	40	16.9
>1 h	4	1.7
They do not define limits	8	3.8
Total	235	100.0

**Table 3:** Investigation of the relationship between participants’ recommendations on exercise in the first trimester of pregnancy and their occupational characteristics.

	**Midwives Recommend Exercise in the First Trimester of Pregnancy**								
			*N*						
			No		Yes	*χ*^2^(1)			*p*
Midwives’ experience in antenatal counseling programs	No	C.	19		44	4.518			0.034
		E.C.	13.7		49.3				
	Yes	C.	14		75				
		E.C.	19.3		69.7				
	**Midwives Recommend Aerobic Exercises in the First Trimester of Pregnancy**								
			*N*						
			No		Yes	*χ*^2^(1)			*p*
Midwives’ experience in antenatal counseling programs	No	C.	21		47				
		E.C.	14.8		53.2	6.090			0.014
	Yes	C.	12		72				
		E.C.	18.2		65.8				
Physician’s professional position		**Physicians Recommend Resistance/Muscle Strengthening Exercises in the First Trimester of Pregnancy**							
			*N*						
			No		Yes			Fisher Exact test	*p*
			C.	E.C.	C.		E.C		
		Director/	16	13.7	4		6.3		
		Curator/							
		Professor						7.123	0.018
		Obstetrics Resident	15	11.7	2		5.3		
		Freelancer	26	31.6	20		14.4		

Note: C.—Count, E.C.—Expected Count.

**Table 4:** Binary logistic regression analyses to explore the relationship between participants’ recommendations of exercise in the first trimester and several independent variables.

**Model A:**	**Healthcare Professionals Recommend Exercise in the first Trimester of Pregnancy**				
	*B*	*S.E.*	*p*	*Exp(B)*	*R* ^2^
Gender (Male)	−0.815	0.360	0.024	0.443	0.132
Believe that exercise during pregnancy is moderately beneficial to pregnant women (“Moderate”—the lowest degree of benefit noted by participants)	−0.512	0.169	0.002	0.599	
Their level of familiarity with international guidelines/recommendations for exercise during pregnancy (“Very”)	0.256	0.103	0.013	1.291	
(Constant)	0.236	0.207	0.254	1.267	
**Model B:**	**Healthcare Professionals Recommend Aerobic Exercise in the First Trimester of Pregnancy**				
	*B*	*S.E.*	*p*	*Exp(B)*	*R* ^2^
Professional healthcare setting (Private sector)	−0.339	0.158	0.032	0.712	
Their level of familiarity with international guidelines/recommendations for exercise during pregnancy (“Very”)	0.233	0.102	0.023	1.262	0.114
The degree of necessity to be informed about guidelines on exercise during pregnancy (“Some/Moderate”—the lowest degrees of necessity noted by participants)	−0.557	0.210	0.008	0.573	
(Constant)	0.166	0.198	0.402	1.180	
**Model C:**	**Healthcare Professionals Recommend Resistance/Muscle-Strengthening Exercises in the First Trimester of Pregnancy**				
	*B*	*S.E.*	*p*	*Exp(B)*	*R* ^2^
Professional healthcare setting (Freelancer)	0.172	0.083	0.039	1.188	
Believe that exercise during pregnancy is moderately beneficial (“Moderate”—the lowest degree of perceived benefit noted by participants)	−0.712	0.346	0.039	0.490	0.091
(Constant)	−1.247	0.233	<0.001	0.287	
**Model D:**	**Healthcare Professionals Recommend Relaxation Exercises in the First Trimester of Pregnancy**				
	*B*	*S.E.*	*p*	*Exp(B)*	*R* ^2^
Believe that exercise during pregnancy is moderately beneficial (“Moderate”—the lowest degree of perceived benefit noted by participants)	−0.535	0.175	0.002	0.585	0.074
(Constant)	0.271	0.151	0.073	1.312	

Note: *B*—Estimated coefficient, *S.E.*—Standard Error, *p*—Statistical significance, *Exp(B)*—odds ratio, *R*^2^—Coefficient of determination.

**Table 5:** Binary logistic regression analyses to explore the relationship between participants’ recommendations to pregnant women on the frequency and duration of exercise in the 1st trimester and various independent variables.

**Model A:**	**Healthcare Professionals Do Not Recommend a Specific Frequency of Exercise in the first Trimester of Pregnancy**				
	*B*	*S.E.*	*p*	*Exp(B)*	*R* ^2^
Professional healthcare setting (Freelancer)	−0.177	0.077	0.022	0.838	0.127
Believe that exercise during pregnancy is moderately beneficial (“Moderate”—the lowest degree of benefit noted by participants)	0.582	0.215	0.007	1.789	
Their level of familiarity with international guidelines/recommendations for exercise during pregnancy (“Very”)	−0.227	0.103	0.027	0.797	
(Constant)	0.749	0.248	0.003	2.116	
**Model B:**	**Healthcare Professionals Recommend 1–3 Days/Week as Exercise Frequency in the First Trimester of Pregnancy**				
	*B*	*S.E.*	*p*	*Exp(B)*	*R* ^2^
Believe that exercise during pregnancy is moderately beneficial (“Moderate”—the lowest degree of benefit noted by participants)	−0.423	0.212	0.046	0.655	0.035
(Constant)	−0.676	0.159	0.000	0.508	
**Model C:**	**Healthcare Professionals Do Not Recommend a Specific Duration of Exercise in the First Trimester of Pregnancy**				
	*B*	*S.E.*	*p*	*Exp(B)*	*R* ^2^
Believe that exercise during pregnancy is moderately beneficial (“Moderate”—the lowest degree of benefit noted by participants)	0.541	0.212	0.011	1.718	0.061
(Constant)	0.322	0.156	0.039	1.380	
**Model D:**	**Healthcare Professionals Recommend 30–45 Min as Duration of Exercise in the First Trimester of Pregnancy**				
	*B*	*S.E.*	*p*	*Exp(B)*	*R* ^2^
Professional healthcare setting (public sector)	0.269	0.103	0.009	1.309	
Their level of familiarity with international guidelines/recommendations for exercise during pregnancy (“Very”)	0.327	0.137	0.017	1.387	0.210
(Constant))	−2.645	0.433	<0.001	0.071	

Note: *B*—Estimated coefficient, *S.E.*—Standard Error, *p*—Statistical significance, *Exp(B)*—odds ratio, *R*^2^—Coefficient of determination.

## Data Availability

All data generated or analyzed during this study will be included in the article as Table(s), Figure(s). Any other data requirement can be directed to the corresponding author upon reasonable request due to the data are not publicly available due to containing information that could compromise the privacy of research participants.
